# Theoretical analysis of the deformation for steel gas pipes taking into account shear effects under surface explosion loads

**DOI:** 10.1038/s41598-022-12698-0

**Published:** 2022-05-23

**Authors:** Tingyao Wu, Hongan Yu, Nan Jiang, Chuanbo Zhou, Xuedong Luo

**Affiliations:** 1 Faculty of Engineering, China University of Geosciences (Wuhan), Wuhan, 430074 Hubei China; 2 CCCC Second Highway Consultants Co., Ltd., Wuhan, 430056 Hubei China

**Keywords:** Engineering, Civil engineering

## Abstract

Ground blast loads are of great importance to the safe operation of steel and gas pipelines, and the results obtained from traditional theoretical formulas for pipeline safety prediction are in error with the actual measured data. In this paper, full-size field tests and corresponding numerical simulations are carried out using Timoshenko beam theory and explosion stress wave theory, which consider shear effects. At the same time, combined with the theory of foundation stiffness and pipeline stiffness flexibility ratio, a modified theoretical model is obtained in line with the actual conditions of the site, which can accurately calculate the deformation and displacement of pipeline underground explosion load, and greatly reduce the error of theoretical prediction results. The innovation of the research results in this paper is that the theoretical stress in the Timoshenko beam can be replaced by the circumferential strain. On the other hand, the modified theoretical solution can obtain the critical weight of explosives to prevent pipeline damage at different buried depths. It provides a theoretical basis for the protection of pipelines’ underground blast loads and provides research ideas for the safe protection and design of pipelines.

## Introduction

As the main way of oil and gas transportation, buried pipelines with different diameters play a huge role in the field of energy transportation, and their structural safety is of great concern^1–3^. However, with the further increase in urbanization, leading to a denser network of buried pipelines, the safety, and protection of pipelines is known as an increasingly important issue^4–6^. In addition, some military operations and civilian production tend to increase the potential for explosive damage to in-service pipelines^7,8^. At the same time, the potential for terrorist attacks has increased in some areas, even with several explosions along oil and gas pipelines^9^. Meanwhile, after a detailed investigation, it was found that in recent years, third-party damage is the main cause of buried pipeline failure and has caused serious accidents^10^. Therefore, it is important to study the damage characteristics of pipelines under blast loading.

For the study of pipelines under blast loads in the past few decades, there have been many experimental and theoretical studies on buried pipelines subjected to ground explosive loads^11–13^. As an example, Zhang et al.^14^ used numerical simulation to study the effects of different factors on pipeline safety, such as the weight of explosives, the horizontal distance of explosives from the pipe, and the burial depth of the pipeline. Song et al.^15^ selected the X70 pipe for field blast testing and obtained four different failure modes based on the deflection and damage level of the pipe, including (a) mode 1 is a large elastic–plastic deformation in the central region; (b) mode 2 is the outer surface of the pipe undergoes large plastic deformation, and becomes thinner in the central region; (c) mode 3 is where both the outer and inner surfaces of the pipe are slightly torn in the central region; (d) mode 4 is where both the front and rear parts of the pipe are completely torn. Based on the work of Mishra et al.^16^ and Zhang et al.^17^, the damage was normalized into local damage criteria and overall failure criteria according to the damage model. On the other hand, the deflection-to-span ratio damage criterion was used to assess the degree of damage to underground pipelines, and the damage to pipelines can be classified into the following four categories, including (a) minor damage; (b) moderate damage; (c) severe damage and (d) collapse. By the example of the work of Bambach et al.^18^, who used laboratory studies to analyze metal beams under transverse blast loading, focusing mainly on the solid metal deformation part. In addition to laboratory studies, theoretical studies are also highly preferred by researchers, some authors such as Abedi et al.^19^ have used a theoretical analytical method to find the beam deflection under the effect of the blast wave. What’s more, Olarewaju et al.^20^ conducted an analytical and numerical study of the static and dynamic response of buried pipelines under blast loading. Meanwhile, some studies have investigated pipe fracture characteristics in addition to simple deformation studies, such as Mirzaei et al.^21^, who analyzed the dynamic fracture of pipes under internal blast loads by numerical simulations and experiments.

It is clear from the above analysis that despite the extensive use of experimental and analytical methods in the literature, numerical simulation analyses cannot be neglected because they can provide valuable information on the response details of structural members with more complex material properties^22–24^. Recently, many scholars have used numerical simulation software such as ABAQUS, LS-DYNA, and AUTODYN to study the effect of blast loading on pipelines, and more detailed dynamic response parameters were obtained, which could not be obtained experimentally^25–27^. However, these pieces of literature are not systematic enough for the study of the dynamic response parameters, and the numerical simulation software has more parameters, in which it is difficult to find many studies containing the correspondence between field tests and numerical simulations. However, it is worth mentioning that the post-damage motion of explosive fragments is very important in the dynamic analysis of structures, while it is difficult to quantify the material properties at the time of deformation and damage, the deformation of pipelines has been difficult to predict so far.

In addition, the research on blasting parameters and pipeline parameters is not comprehensive enough, and there is not enough theoretical explanation on how to apply dynamic response characteristics to pipeline safety protection. More importantly, many of the methods used to analyze the numerical simulation response data are not sufficiently comprehensive and accurate, and the failure modes of buried pipelines of different diameters and burial depths under surface explosive loads have not been adequately investigated. In addition, most of the numerical simulations are commercial software and the research results are similar. Therefore, this paper introduces a theoretical analysis approach, which has the advantage that the safety criterion model for pipelines under different surface explosive loads can be studied by combining the theoretical analysis approach with damage discrimination criteria.

On the basis of the above analysis, this paper considers the impact of surface explosion load on the pipeline, at the same time, multiple dynamic response parameters are analyzed, and the buckling damage of the buried pipeline is evaluated based on the theoretical analysis method. The field tests and numerical calculation model on full-size X42 (L290) steel pipes were designed and carried out under surface explosive loads. Meanwhile, the relevant parameters of the numerical model were well verified by comparing the field data with the numerical simulation data. At the same time, combined with Timoshenko beam theory considering the shear effect and flexible ratio theory of foundation stiffness to pipeline stiffness, the vertical displacement prediction equation of pipeline is modified. More importantly, the field tests were generally well reproduced, which also further investigated the flexural damage of buried pipelines under the action of the ground blast. Finally, the effects of burial depth and explosive level on different dynamic characteristics of the pipeline are discussed in conjunction with the least-squares approach, and the maximum vertical displacement of the pipeline is well predicted, and the theoretical analysis results reflect the key phenomena well.

## Material and methods

### Theoretical model calculation method for initial impact pressure

When the explosive is detonated, the blast stress wave will be along the free surface of the direction of rapid propagation impact around the rock body. When the blast stress wave reaches the interface between air and rock, the external force acting on the rock interface is the initial impact pressure. Many impact pressure equations^28–30^ for explosives have been introduced and the initial impact pressure can be used to express the pressure of the blast stress wave, as shown in Eq. (1):

1 $$ P_{{0}} = \frac{{\rho_{{0}} D^{2} }}{{2\left( {\beta + 1} \right)}} $$

where *P*_0_ is the role of the explosion pressure, *ρ*_0_ is the density of the explosion, *D* is the speed of the explosion, *β* is the adiabatic expansion index of the products of the explosion, is taken as 3.0. When the surface explosive is detonated, the blast shock wave and stress wave will produce a fragmentation zone, fracture zone, and elastic zone around the center of the explosive. The values of the shock and stress waves decrease as the blast time increases. The curves of shock and stress waves with the blast time are in the form of an exponential function. The attenuation coefficient is as shown in Eqs. (2)–(3).

2 $$ m_{1} = 2 + \frac{{\mu_{s} }}{{1 - \mu_{s} }} $$

3 $$ m_{2} = 2 - \frac{{\mu_{s} }}{{1 - \mu_{s} }} $$

where *μ*_*s*_ is the Poisson's ratio of the soil. The stress wave reaches the outer edge of the fracture zone and then enters the elastic zone. In the elastic zone, the stress wave action only produces elastic vibration, the explosion pressure as shown in Eq. (4):

4 $$ p_{e} = \frac{{\rho_{{\text{e}}} D^{2} }}{2(\gamma + 1)}\left( {\frac{{r_{{\text{c}}} }}{{r_{{\text{b}}} }}} \right)^{{ - m_{1} }} \left( {\frac{{r_{{\text{f}}} }}{{r_{{\text{c}}} }}} \right)^{{ - m_{2} }} $$

where *P*_*e*_ is the explosion pressure at the boundary of the elastic zone, *r*_*b*_ is the radius of the package; *r*_*c*_ and *r*_*f*_ are the radius of the fracture zone and the radius of the elastic zone, respectively, the radius of the fracture zone caused by conventional explosives *r*_*c*_ is 3–5 times the radius of the package, the radius of the elastic zone *r*_*f*_ is 10–15 times the radius of the package^31^, in this paper, *r*_*c*_ = 3*r*_*b*_, *r*_*f*_ = 11*r*_*b*_.

Assuming that the stress wave propagates to the peak explosion pressure at point *G* of the pipe is *P*_*G*_:

5 $$ p_{G} = p_{e} \left( {\frac{{r_{1} }}{{r_{f} }}} \right)^{{ - m_{2} }} $$

6 $$ r_{1} = \sqrt {l_{1}^{2} + {\text{z}}^{2} } $$

**Figure 1:**
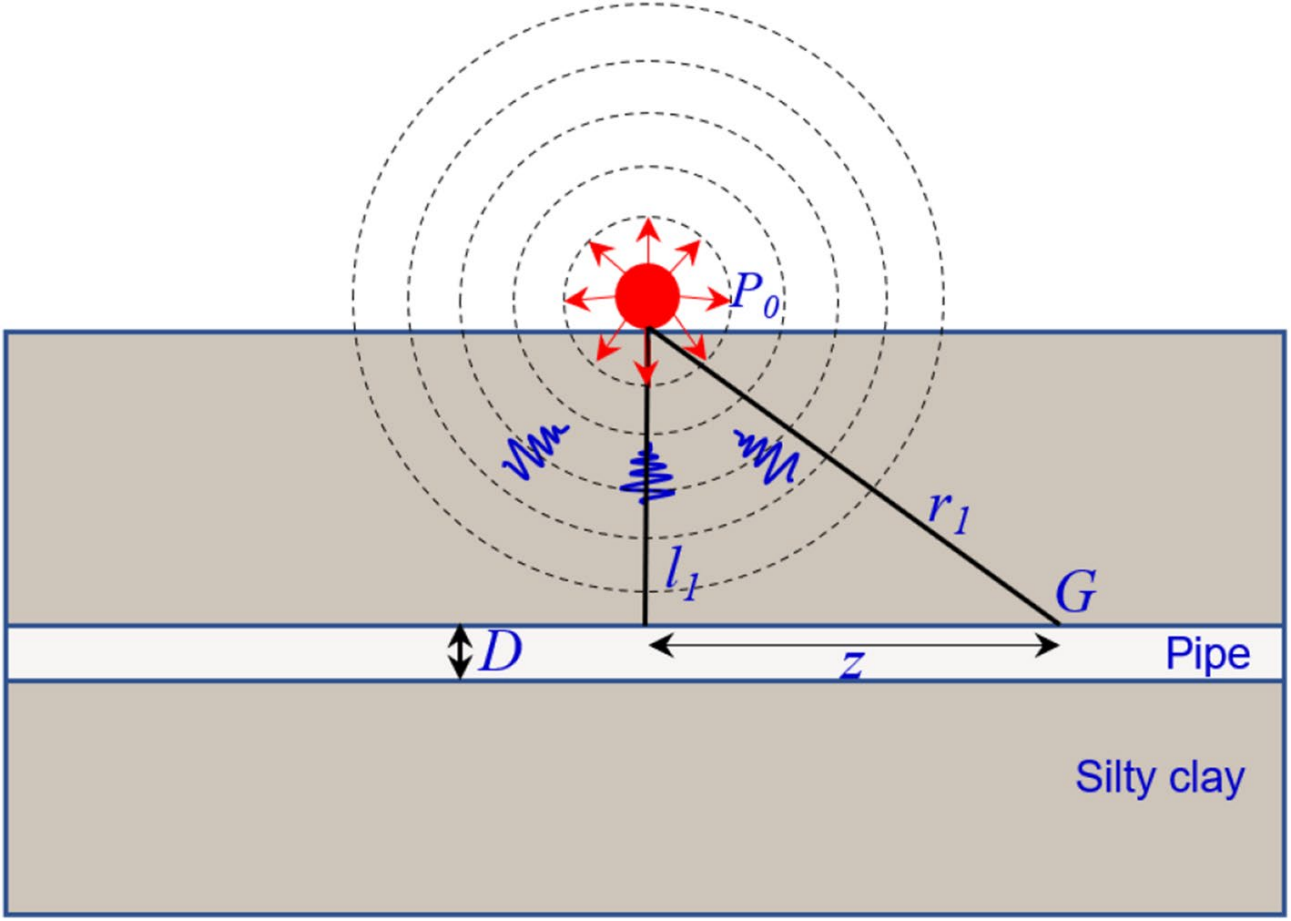
Schematic diagram of the forces on the pipe.

Due to the symmetry of the pipe with the blast load, only half of the load acting on the pipe is considered. Also ignoring the axial load component of the pipe (*Z* direction in Fig. 1), the blast load *q(x)* acting on the pipe can be expressed as Eq. (7):

7 $$ q\left( x \right) = \frac{\pi D}{2}p_{e} \left( {\frac{{r_{1} }}{{r_{f} }}} \right)^{{ - m_{2} }} \frac{{l_{1} }}{{r_{1} }} $$

### Theory of longitudinal deformation of pipes considering shear effects

**Figure 2:**
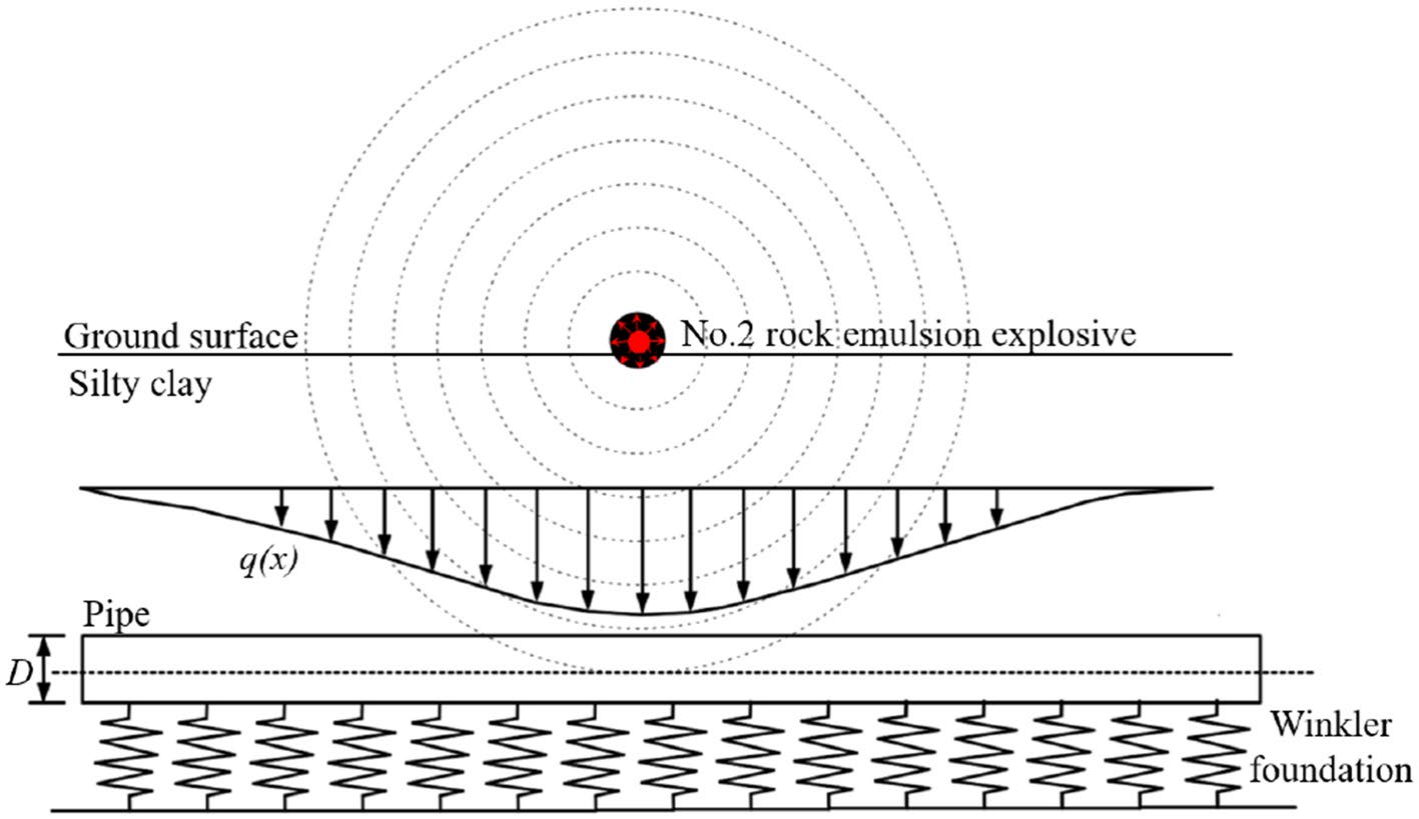
Calculation model for the impact on the pipeline.

**Figure 3:**
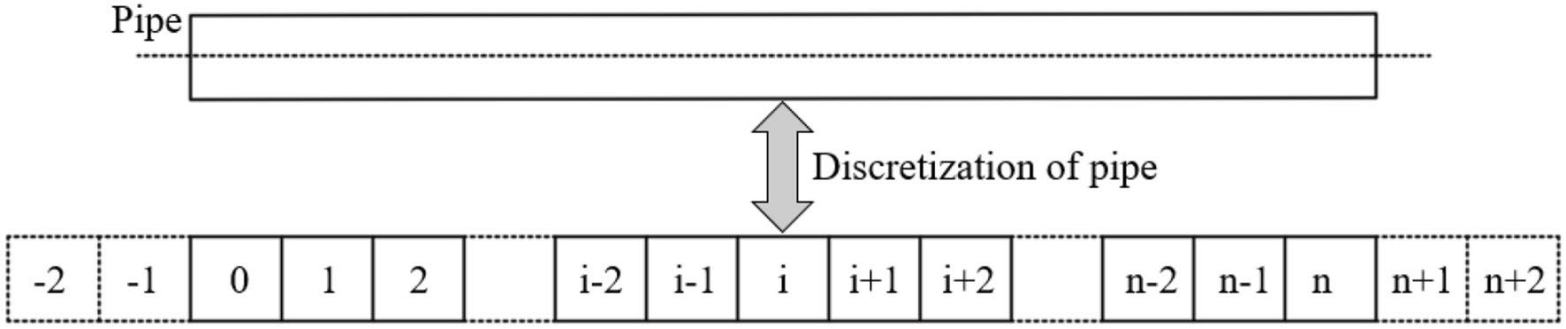
Discrete diagram of the pipe.

As mentioned above we have mainly discussed the propagation characteristics of blast stress waves in the soil. When the surface of a pipe is subjected to a transverse shock wave, the deformation of the pipe belongs to the dynamic bending problem of the pipe, which can be described by the treatment of vibration or by the treatment of fluctuations, where the fluctuations are called bending waves. Meanwhile, bending waves are the consequence of the joint coupling of interdependent bending moment perturbations and shear perturbations, in which the shear effect is already included. In contrast to the role of shear in the quasi-static response of a pipe, the study of the dynamic response of a pipe subjected to transverse impact loads plays a more important role due to the inclusion of transverse inertia shear in the set of control equations. The explosion of the upper ground surface causes a concentration of stress, which in turn causes bending and deformation of the pipe below. To simplify the calculations, the following assumptions are made in the calculation model in this paper: (1) the pipe is assumed to be a Timoshenko beam with shear effects; (2) pipe–ground interaction is considered through the Winkler foundation model; (3) soft ground creep and drainage consolidation are not considered. The calculation model was solved using the commonly used two-stage analysis method^32–34^. Firstly, the additional distributed load on the pipe caused by surface explosive blasting is calculated by the blast stress wave mechanics equation; secondly, the finite difference method is used to establish the analytical solution for the longitudinal deformation of the pipe under the additional distributed load. Figure 3 shows the model for calculating the effect of the surface blast on an underlying pipe. The surface explosive blast results in a concentration of stress in the existing ground, which causes an additional vertical distributed load *q(x)* to be applied to the pipe, as shown in Fig. 2. The additionally distributed load *q(x)* is obtained by solving for "Theoretical model calculation method for initial impact pressure" section.

Under the additional load *q(x)*, the equilibrium differential equation for the Timoshenko beam on Winkler foundations concerning vertical displacement *w(x)* and angle of rotation θ can be obtained as shown in Eq. (8).

8a $$ - \frac{{\text{d}}}{{{\text{d}}x}}\left\{ {(k^{{\prime }} GA)_{{{\text{eq}}}} \left[ {\frac{{{\text{d}}w(x)}}{{{\text{d}}x}} - \theta } \right]} \right\} + q(x)D_{{\text{t}}} = kD_{{\text{t}}} w(x) $$

8b $$ (EI)_{{{\text{eq}}}} \frac{{{\text{d}}^{2} \theta }}{{{\text{d}}x^{2} }} + (k^{{\prime }} GA)_{{{\text{eq}}}} \left[ {\frac{{{\text{d}}w(x)}}{{{\text{d}}x}} - \theta } \right] = 0 $$

where: *EI*_*eq*_ is the longitudinal equivalent bending stiffness of the pipe, *(κ′GA)*_*eq*_ is the equivalent shear stiffness of the pipe, *D*_*t*_ is the diameter of the pipe, *k* is the foundation reaction coefficient, and *q(x)* is the additional load caused by the surface explosion.

Where* t* is the wall thickness of pipe:

9 $$ (EI)_{eq} = EI\quad I = \frac{{t^{3} }}{12} $$

For the vertical bearing soil springs, based on the work of the Federal Emergency Management Agency (FEMA) and the American Society of Civil Engineers (ASCE)^35^, the maximum vertical upward soil bearing capacity *Q*_*d*_ can be obtained.

10a $$ Q_{d} = \Delta_{qd} \cdot k^{{\prime }} = N_{c} cD + N_{q} \gamma^{{\prime }} HD_{{\text{t}}} + N_{\gamma } \gamma \frac{{D^{2} }}{2} $$

where: *Nc, Nq, N*_*γ*_ are bearing capacity factors, *γ* is the total unit weight of soil, γ′ is the effective unit weight of soil, ∆_*qd*_ is vertical displacement to develop *Q*_*d*_, c is soil cohesion, *D*_*t*_ is the diameter of the pipe, *H* is the depth of cover to the pipe centerline.

10b $$ Nc = [\cot (\varphi + 0.001)]\left\{ {\exp [\pi \tan (\varphi + 0.001)]\tan^{2} \left( {45 + \frac{\varphi + 0.001}{2}} \right) - 1} \right\} $$

10c $$ N_{q} = \exp (\pi \tan \varphi )\tan^{2} \left( {45 + \frac{\varphi }{2}} \right)\quad N_{\gamma } \quad = \quad e^{(0.18\varphi - 2.5)} $$

where ∆_*qd*_ is displacement at *Q*_*d*_, ∆_*qd*_ = 0.2*D*.

Decoupling Eq. (8), we can obtain the differential equations for the vertical displacement *w*(*x*) and the angle of rotation *θ*, respectively.

11a $$ \frac{{{\text{d}}^{4} w(x)}}{{{\text{d}}x^{4} }} + \frac{{kD_{{\text{t}}} }}{{(\kappa GA)_{{{\text{eq}}}} }}\frac{{{\text{d}}^{2} w(x)}}{{{\text{d}}x^{2} }} - \frac{{kD_{{\text{t}}} }}{{(EI)_{{{\text{eq}}}} }}w(x) = - \frac{1}{{(EI)_{{{\text{eq}}}} }}q(x)D_{{\text{t}}} + \frac{{D_{{\text{t}}} }}{{(\kappa GA)_{{{\text{eq}}}} }}\frac{{{\text{d}}^{2} q(x)}}{{{\text{d}}x^{2} }} $$

11b $$ \theta = \frac{{{\text{d}}w(x)}}{{{\text{d}}x}} + \frac{1}{{(\kappa GA)_{{{\text{eq}}}} }}\int {\left[ {kD_{{\text{t}}} w(x) - q(x)D_{{\text{t}}} } \right]} {\text{d}}x $$

The longitudinal deformation of the existing pipe under the additional load *q(x)* caused by the blasting of the explosive at the surface can be obtained by solving Eq. (11a). As Eq. (11a) is a fourth-order ordinary differential equation, there is some difficulty in solving it mathematically. To simplify the calculation, the finite difference method is used to solve the equation. Figure 3 shows the discrete diagram of the pipe. The pipe is discretized into n + 5 nodal units (including 2 dummy nodal units at the ends of the pipe), each with a length of *l* (0.2 m). According to the standard finite difference principle, the finite difference form of the differential terms of Eq. (11a) is as follows.

12a $$ \frac{{{\text{d}}^{4} w(x)}}{{{\text{d}}x^{4} }} = \frac{{6w_{i} - 4\left( {w_{i + 1} + w_{i - 1} } \right) + \left( {w_{i + 2} + w_{i - 2} } \right)}}{{l^{4} }} $$

12b $$ \frac{{{\text{d}}^{2} w(x)}}{{{\text{d}}x^{2} }} = \frac{{w_{i + 1} - 2w_{i} + w_{i - 1} }}{{l^{2} }} $$

12c $$ \frac{{{\text{d}}^{2} q(x)}}{{{\text{d}}x^{2} }} = \frac{{q_{i + 1} - 2q_{i} + q_{i - 1} }}{{l^{2} }} $$

where: *w*_*i*_ is the vertical displacement of nodal unit *i*, and *q*_*i*_ is the additional load at nodal unit *i*. Substituting Eqs. (12a)–(12c) into Eq. (11a), the finite difference expression for the Timoshenko beam on Winkler foundations is obtained as follows in Eq. (13).

13 $$ \begin{aligned} & \frac{{6w_{i} - 4\left( {w_{i + 1} + w_{i - 1} } \right) + \left( {w_{i + 2} + w_{i - 2} } \right)}}{{l^{4} }} - \frac{{kD_{{\text{t}}} }}{{(\kappa GA)_{{{\text{eq}}}} }}.\frac{{w_{i + 1} - 2w_{i} + w_{i - 1} }}{{l^{2} }} + \frac{{kD_{{\text{t}}} }}{{(EI)_{{{\text{eq}}}} }}w_{i} \\ & \quad = \frac{1}{{(EI)_{{{\text{eq}}}} }}q_{i} D_{{\text{t}}} - \frac{{D_{{\text{t}}} }}{{(\kappa GA)_{{{\text{eq}}}} }}\frac{{q_{i + 1} - 2q_{i} + q_{i - 1} }}{{l^{2} }} \\ \end{aligned} $$

Assuming that the pipe is free at both ends, the bending moment *M* and the shear force *Q* at both ends of the pipe are 0.

14a $$ M_{0} = M_{n} = - (EI)_{{{\text{eq}}}} \frac{{{\text{d}}\theta }}{{{\text{d}}x}} = 0 $$

14b $$ Q_{0} = Q_{n} = - (\kappa GA)_{{{\text{eq}}}} \left( {\frac{{{\text{d}}w}}{{{\text{d}}x}} - \theta } \right) = 0 $$

Equation (15) can be obtained from Eq. (8b).

15 $$ \frac{{{\text{d}}w(x)}}{{{\text{d}}x}} - \theta = - \frac{{(EI)_{{{\text{eq}}}} }}{{(\kappa GA)_{{{\text{eq}}}} }}\frac{{{\text{d}}^{2} \theta }}{{{\text{d}}x^{2} }} $$

Taking one derivative of Eqs. (8a), (16) and (17) can be obtained.

16 $$ - (\kappa GA)_{{{\text{eq}}}} \frac{{{\text{d}}^{3} w(x)}}{{{\text{d}}x^{3} }} + (\kappa GA)_{{{\text{eq}}}} \frac{{{\text{d}}^{2} \theta }}{{{\text{d}}x^{2} }} + D_{{\text{t}}} \frac{{{\text{d}}q(x)}}{{{\text{d}}x}} = kD_{{\text{t}}} \frac{{{\text{d}}w(x)}}{{{\text{d}}x}} $$

17 $$ \frac{{{\text{d}}^{2} \theta }}{{{\text{d}}x^{2} }} = \frac{{{\text{d}}^{3} w(x)}}{{{\text{d}}x^{3} }} + \frac{{kD_{{\text{t}}} }}{{(\kappa GA)_{{{\text{eq}}}} }}\frac{{{\text{d}}w(x)}}{{{\text{d}}x}} - \frac{{D_{{\text{t}}} }}{{(\kappa GA)_{{{\text{eq}}}} }}\frac{{{\text{d}}q(x)}}{{{\text{d}}x}} $$

Combining Eqs. (15)–(17), (14b) can be written as Eq. (18).

18 $$ Q_{0} = Q_{n} = (EI)_{{{\text{eq}}}} \left[ {\frac{{{\text{d}}^{3} w(x)}}{{{\text{d}}x^{3} }} + \frac{{kD_{{\text{t}}} }}{{(\kappa GA)_{{{\text{eq}}}} }}\frac{{{\text{d}}w(x)}}{{{\text{d}}x}} - \frac{{D_{{\text{t}}} }}{{(\kappa GA)_{{{\text{eq}}}} }}.\left. {\frac{{{\text{d}}q(x)}}{{{\text{d}}x}}} \right] = 0} \right. $$

Taking the differential form of Eq. (18), the differential expression for the shear force *Q* at each end of the pipe can be obtained as shown in Eq. (19).

19a $$ Q_{0} = (EI)_{{{\text{eq}}}} \left[ {\frac{{w_{2} - 2w_{1} + 2w_{ - 1} - w_{ - 2} }}{{2l^{3} }} + \frac{{kD_{{\text{t}}} }}{{(\kappa GA)_{{{\text{eq}}}} }}.\left. {\frac{{w_{1} - w_{ - 1} }}{2l} - \frac{{D_{{\text{t}}} }}{{(\kappa GA)_{{{\text{eq}}}} }}\frac{{q_{1} - q_{ - 1} }}{2l}} \right] = 0} \right. $$

19b $$ Q_{n} = (EI)_{{{\text{eq}}}} \left[ {\frac{{w_{n + 2} - 2w_{n + 1} + 2w_{n - 1} - w_{n - 2} }}{{2l^{3} }} + \frac{{kD_{{\text{t}}} }}{{(\kappa GA)_{{{\text{eq}}}} }}.\left. {\frac{{w_{n + 1} - w_{n - 1} }}{2l} - \frac{{D_{t} }}{{(\kappa GA)_{{{\text{eq}}}} }}\frac{{q_{n + 1} - q_{n - 1} }}{2l}} \right] = 0} \right. $$

Equation (20) can be obtained from Eq. (8a).

20 $$ \frac{{{\text{d}}\theta }}{{{\text{d}}x}} = \frac{{{\text{d}}^{2} w(x)}}{{{\text{d}}x^{2} }} + \frac{{kD_{{\text{t}}} w(x)}}{{(\kappa GA)_{{{\text{eq}}}} }} - \frac{{q(x)D_{{\text{t}}} }}{{(\kappa GA)_{{{\text{eq}}}} }} $$

After substituting Eq. (20) into Eq. (17), we can obtain Eq. (21).

21a $$ M_{0} = - (EI)_{{{\text{eq}}}} \left[ {\frac{{w_{1} - 2w_{0} + w_{ - 1} }}{{l^{2} }} + \frac{{kD_{{\text{t}}} }}{{(\kappa GA)_{{{\text{eq}}}} }}w_{0} - \left. {\frac{{q_{0} D_{{\text{t}}} }}{{(\kappa GA)_{{{\text{eq}}}} }}} \right] = 0} \right. $$

21b $$ M_{n} = - (EI)_{{{\text{eq}}}} \left[ {\frac{{w_{n + 1} - 2w_{n} + w_{n - 1} }}{{l^{2} }} + \frac{{kD_{{\text{t}}} }}{{(\kappa GA)_{{{\text{eq}}}} }}w_{n} - \left. {\frac{{q_{n} D_{{\text{t}}} }}{{(\kappa GA)_{{{\text{eq}}}} }}} \right] = 0} \right. $$

Equation (22) is obtained from Eqs. (21a)–(21b).

22a $$ w_{ - 1} = \left[ { - \frac{{kD_{{\text{t}}} }}{{(\kappa GA)_{{{\text{eq}}}} }}l^{2} + 2} \right]w_{0} - w_{1} + \frac{{q_{0} D_{{\text{t}}} }}{{(\kappa GA)_{{{\text{eq}}}} }}l^{2} $$

22b $$ w_{n + 1} = \left[ { - \frac{{kD_{{\text{t}}} }}{{(\kappa GA)_{{{\text{eq}}}} }}l^{2} + 2} \right]w_{n} - w_{n - 1} + \frac{{q_{n} D_{{\text{t}}} }}{{(\kappa GA)_{{{\text{eq}}}} }}l^{2} $$

Substituting Eqs. (22a)–(22b) into Eqs. (19a)–(19b), we can obtain Eq. (23).

23 $$ \begin{aligned} w_{ - 2} & = \left[ { - \frac{{4kD_{{\text{t}}} }}{{(\kappa GA)_{{{\text{eq}}}} }}l^{2} + 4 + \frac{{D_{{\text{t}}}^{2} k^{2} }}{{(\kappa GA)_{{{\text{eq}}}}^{2} }}l^{4} } \right]w_{0} - \left[ {\frac{{2kD_{{\text{t}}} }}{{(\kappa GA)_{{{\text{eq}}}} }}l^{2} - 4} \right]w_{1} + w_{2} \\ & \quad + \left[ { - \frac{{kD_{{\text{t}}}^{2} l^{4} }}{{(\kappa GA)_{{{\text{eq}}}}^{2} }} + \frac{{2D_{{\text{t}}} l^{2} }}{{(\kappa GA)_{{{\text{eq}}}} }}} \right]q_{0} - \frac{{D_{{\text{t}}} }}{{(\kappa GA)_{{{\text{eq}}}} }}l^{2} \left( {q_{1} - q_{ - 1} } \right) \\ \end{aligned} $$

Equation (13) can be written in matrix form as shown in Eq. (24).

24 $$ \left( {\left[ {K_{1} } \right] - \left[ {K_{2} } \right] + \left[ {K_{3} } \right]} \right)\{ w\} = \left[ {Q_{1} } \right] - \left[ {Q_{2} } \right] - \left[ {Q_{3} } \right] $$

where: [*K*_1_] is the displacement stiffness matrix of the pipe, [*K*_2_] is the shear stiffness matrix of the pipe, [*K*_3_] is the flexural stiffness matrix of the pipe, {*w*} is the vertical displacement column vector of the pipe, *Q*_*1*_ is the additional load column vector of the pipe, *Q*_*2*_ is the load correction column vector of the pipe, and *Q*_*3*_ is the supplementary vector of the pipe for the solution.

### Field tests

**Figure 4:**
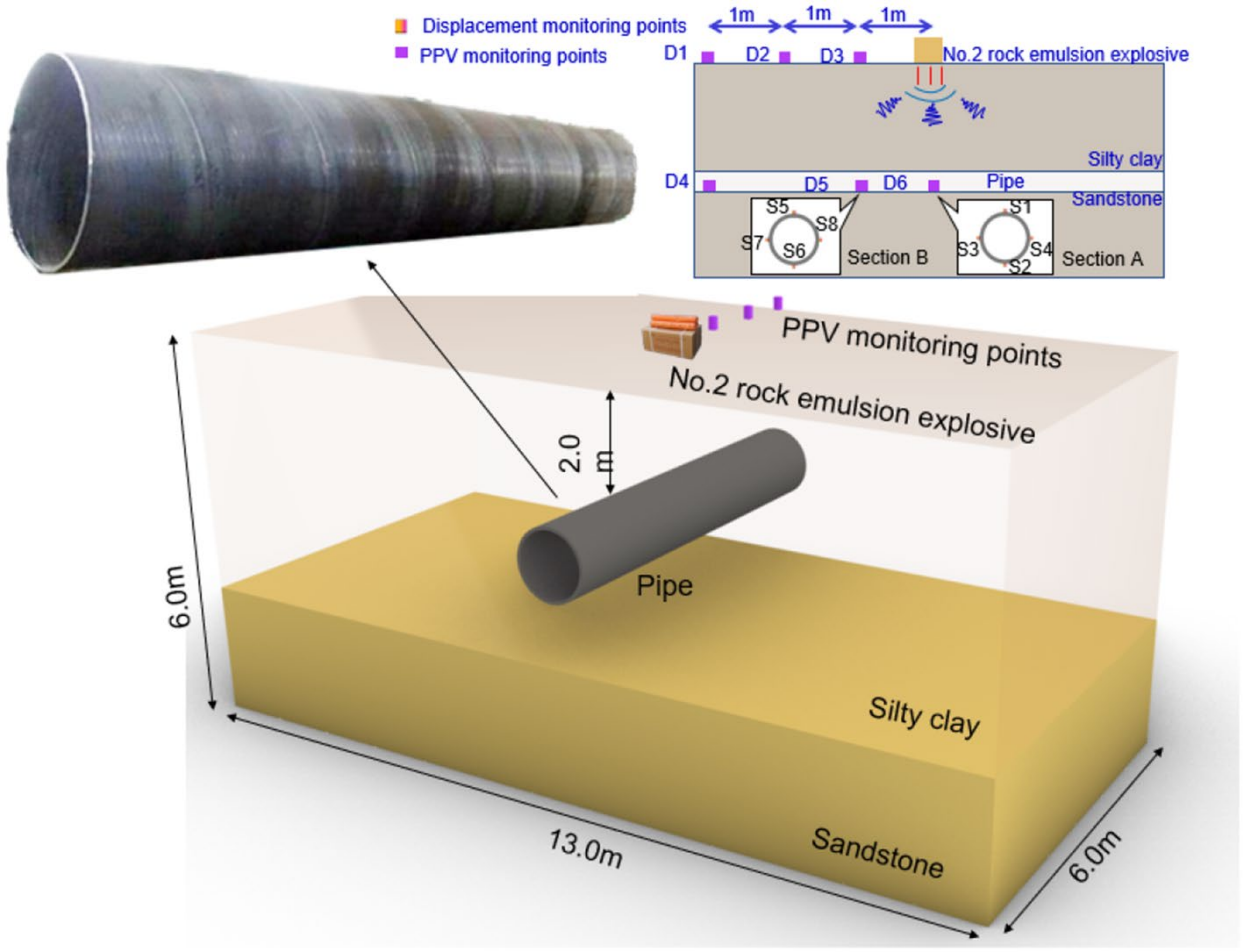
Schematic diagram of the full-scale field tests.

**Figure 5:**
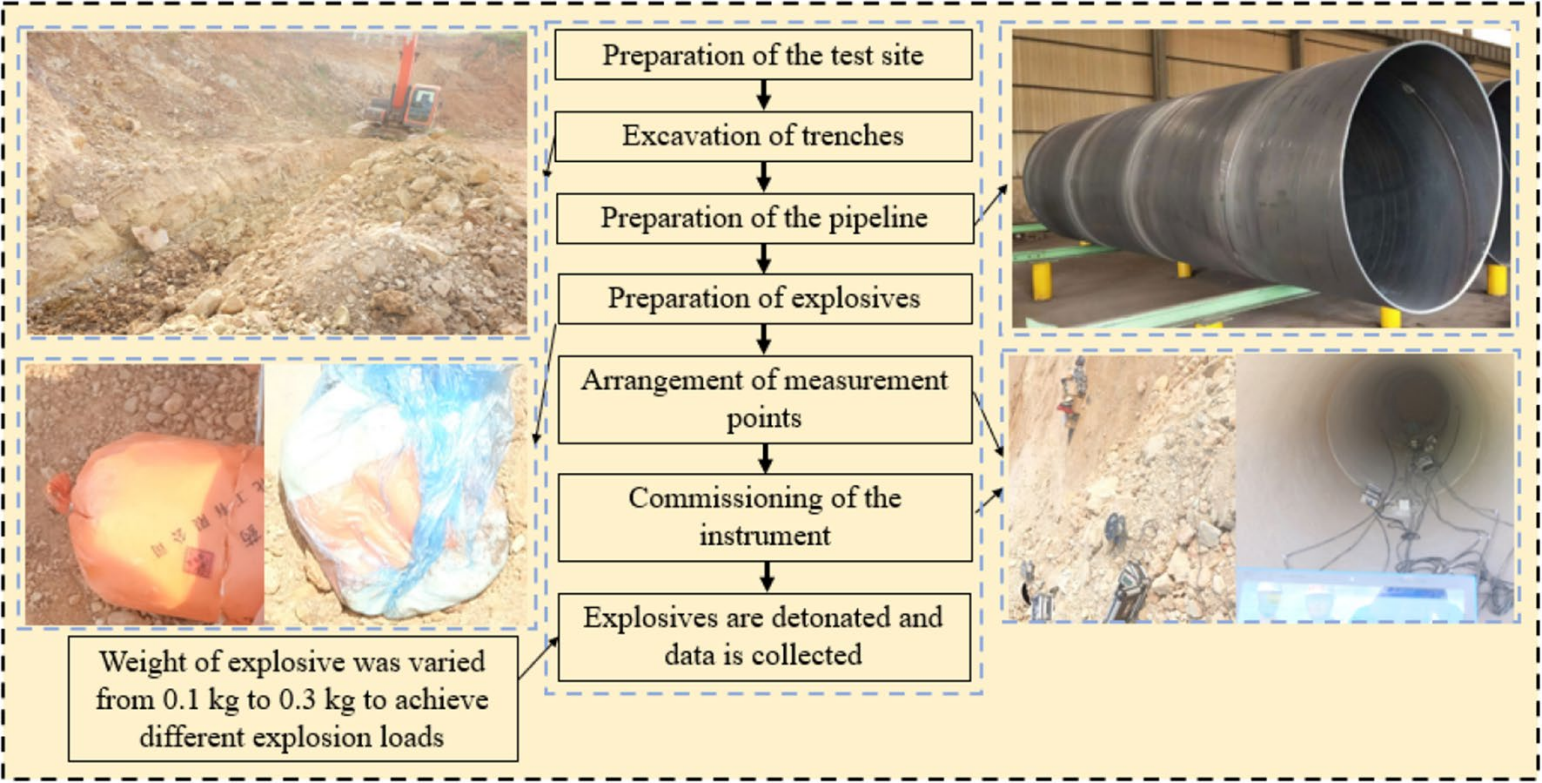
Procedure of the field tests.

The full-scale field tests were conducted at the field test site where the explosives were placed on the ground surface directly above the center of the pipe, and the sketch of the experimental setup is shown in Fig. 4. The explosion load was caused by the explosion of No.2 rock emulsion explosive, in the study of field testes, the weight of the explosive was varied from 0.1 to 0.3 kg to achieve different explosion loads, in which the peak particle velocity (PPV) and displacement of both the pipe and the surface were recorded. The full-scale field tests scheme is shown in Table 1. The field test procedure is shown in Fig. 5.

**Table 1:** Scheme of full-scale field tests.

Number of test case	1	2	3	4	5
Weight of explosives/kg	0.1	0.15	0.2	0.25	0.3

### Computational model

**Figure 6:**
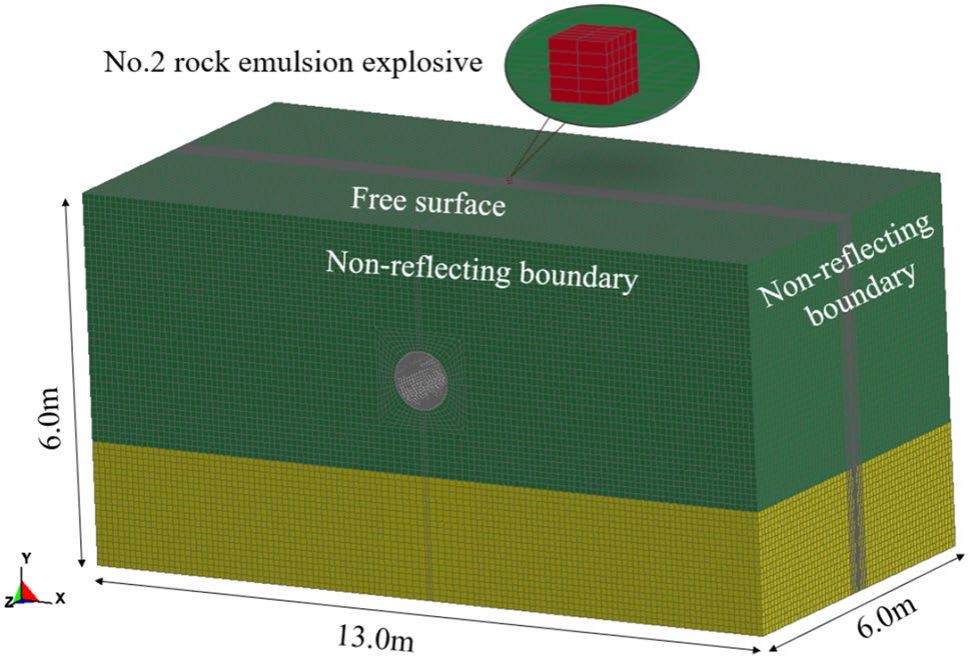
Schematic diagram of the computational model.

Explosion mechanics is a nonlinear problem, which is very difficult to study the buckling damage of buried pipes subjected to surface explosive loads using analytical calculations. Therefore, the numerical simulation is more suitable to solve this problem^36^. The schematic diagram of the computational model is shown in Fig. 6. In this paper, the inner diameter of the steel pipe is 1000 mm with a wall thickness of 10 mm, which is a typical size of an oil and gas transmission pipeline. The type of steel pipe is X42(L290), which means its yield stress is 290 MPa^37^. Meanwhile, the burial depth of the pipe is the same as the field test, and the No. 2 rock emulsion blast load is used to simulate the ground explosion. The dimensions of the whole computational model are 10 m × 4.8 m × 6.0 m in the *X*, *Y*, and *Z* directions, respectively.

In this paper, the behavior of materials is modeled using various nonlinear material models and equations of state. These material models and equations of state are briefly described below. Before the explosion is detonated, the material model for the No. 2 rock emulsion explosive can be described as HIGH_EXPLOSIVE_BURN. After the explosion, the No. 2 rock emulsion explosive exhibits gaseous properties and it is modeled using the JWL equation of state, which describes the pressure resulting from the expansion of the chemical explosive explosion products. It can be written as shown in Eq. (25) below^38^.

25 $$ p = A\left( {1 - \frac{\omega }{{R_{1} V_{1} }}} \right)e^{{ - R_{1} V_{1} }} + B\left( {1 - \frac{\omega }{{R_{2} V_{1} }}} \right)e^{{ - R_{2} V_{1} }} + \frac{{\omega E_{1} }}{{V_{1} }} $$

where *p* is the pressure of the explosive, *E*_1_ is the internal energy per unit volume of explosive, and *V*_1_ is the relative volume of the explosive. The values of the constants for many common explosives, such as *A*, *B*, *R*_1_, *R*_2*,*_ and ω, are determined by dynamic experiments. The values used for the explosive state parameters and the *JWL* equation are listed in Table 2.

**Table 2:** Parameters of explosives materials.

Density/(g/cm^3^)	Velocity of detonation/(m/s)	Pressure of detonation/GPa	A/GPa	B/GPa	R_1_	R_2_	E_0_/GPa
1.09	3600	3.24	234.4	0.182	4.2	0.9	4.192

The mechanical behavior of soils and rocks is described by the *MAT_PLASTICITY_POLYMER material model, the physical parameters of the soil and rock are shown in Table 3.

**Table 3:** Physical and mechanical parameters of steel pipeline and soil and rock.

Type	*γ′*/(N m^−3^)	*γ*/(N m^−3^)	*E*/GPa	*G*/GPa	*μ*	*C*/MPa	*φ*/(°)	*σ*_*s*_/MPa
Steel pipe	9500	–	210	79	0.35	–	–	290
Silty clay	19,800	25,000	0.62	4.3	0.35	0.65	25	–
Sandstone	26,800	–	52.00	11.2	0.25	5.500	43	2.580

The solid elements are applied to the explosives, soil, rock, and pipeline areas. The interface between the pipe and other materials is simulated using a fluid–solid coupling algorithm. Except for the free surface at the top of the air zone, the infinite layer is simulated using non-reflective boundary conditions to avoid reflective wave perturbation results.

## Results

### Model validation

**Figure 7:**
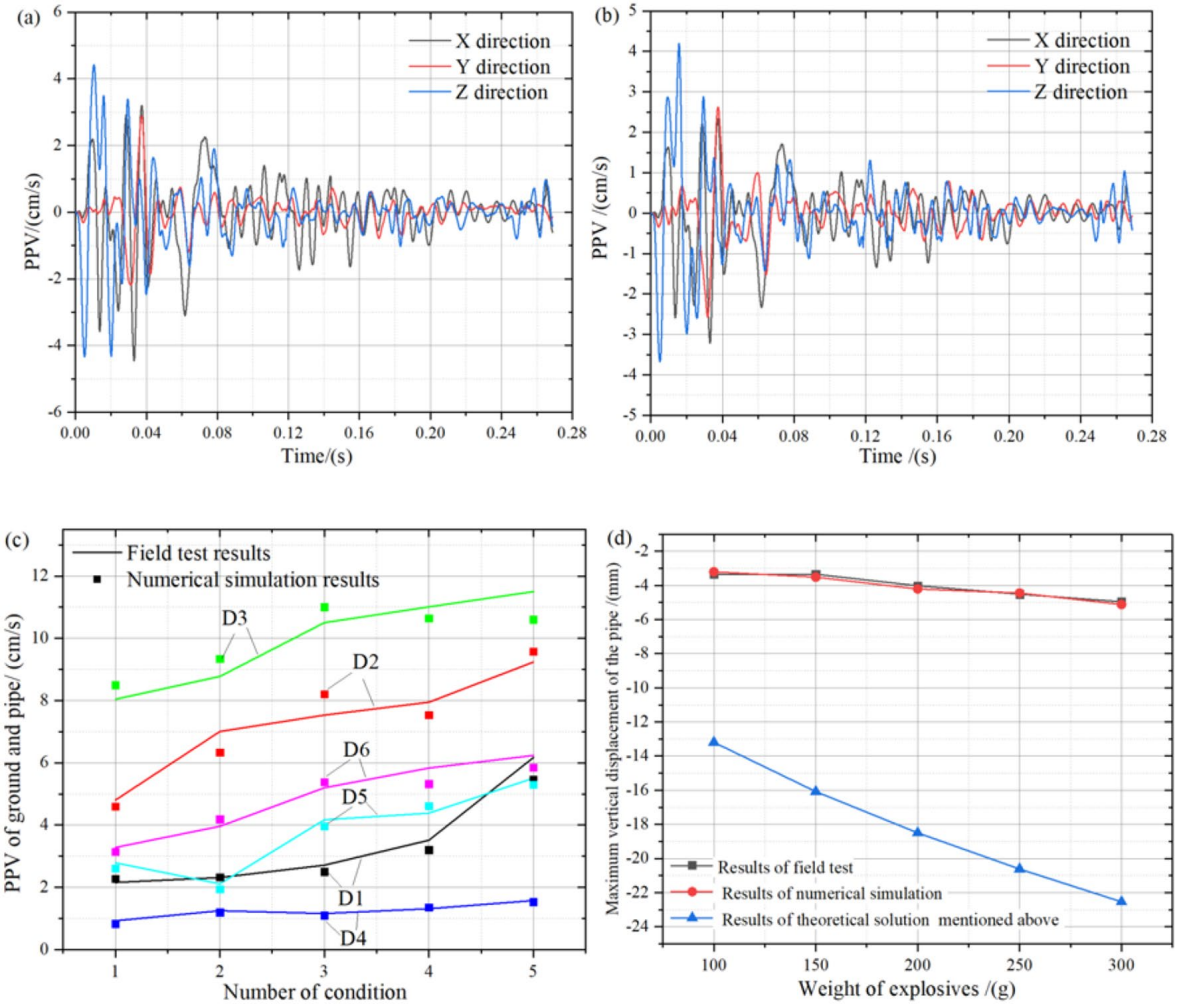
Comparison of field measured data and numerical simulation: (**a**) field test results of *D1*, (**b**) numerical simulation results of *D1*, (**c**) PPV of ground and pipe, (**d**) comparison of results of the field test and numerical simulation with a theoretical solution.

As shown in Table 1, the different weights of No. 2 rock emulsion explosives were placed on the ground surface directly above the pipe, and the PPV of the pipe and ground surface were counted and compared with the above numerical simulation results, as shown in Fig. 7. The velocity of the pipe increased as the weight of the explosive increased, and most of the numerical simulation results were smaller than the field test results, meanwhile, the waveform variation of the vibration velocity at the same time is consistent. This is because the influence of soil joints on wave propagation is not considered in numerical simulation, and the refraction and reflection of wave joints in the soil increases the amplitude of waves on the pipeline surface. At the same time, it is easy to see from the waveform of the model that the variation trend between the two curves is consistent, indicating that the parameters of the numerical model are in line with the actual engineering practice. Also, on closer inspection of Fig. 7c, it is not difficult to find that the error between the numerical simulation results and the field test results decreases as the weight of the explosive increases. This is because with the increase of the weight of explosives, the velocity amplitude of blasting vibration wave becomes larger and larger, and the velocity error on the pipeline caused by the wave's reflection becomes smaller and smaller gradually. Therefore, the parameters of the numerical model are available to study the buckling effect of the pipe under the surface explosion load, while the numerical simulation results are more accurate and reliable when the weight of the explosive is larger.

However, based on the maximum vertical displacement of the pipe in Fig. 7d, it can be seen that the numerical simulation results are very close to the field test results, while on the contrary, the difference between the theoretical results and the other two curves is surprisingly large. This is because a certain parameter in the theoretical model leads to large stress on the pipeline, resulting in a large displacement of the pipeline. After comparing with the field data, it is not difficult to find that such a result is not accurate, so the force acting on the pipeline should be further analyzed and solved. Therefore, the theoretical solution process needs to be further modified before it can meet the need to fit the safety prediction equation of the pipeline regarding the weight of explosives in the actual project.

## Discussion

### Correction of theoretical equations

The relative stiffness of the pipe to its embedded medium affects the stress concentration in the restrained structure. For all practical purposes, a restrained structure should be considered fully flexible when the ratio of the flexibility of the pipe to the flexibility of the ground is greater than 10^39^, which is named the flexible ratio theory of the foundation stiffness to the pipe stiffness. For pipelines, the flexibility ratio J of the foundation stiffness to the pipe stiffness is defined by Eq. (26):

26 $$ J = \frac{{E_{s} /(1 + \mu_{s} )}}{{\left[ {6EI_{L} /\left( {1 - \mu_{L}^{2} } \right)} \right]\left( {1/r_{L}^{3} } \right)}} $$

where *E*_*s*_ and *E* are Young's modulus of the ground and the pipe, *μ*_*s*_ and *μ*_L_ are the Poisson's ratio of the ground and the pipe respectively, *r* is the radius of the pipe and *I* is the rotational inertia of the pipe.

Combining Eq. (26) we can obtain Eq. (27):

27 $$ J = \frac{{E_{s} \left( {1 - \nu_{L}^{2} } \right)D_{t}^{3} }}{{4Et^{3} (1 + \nu )}} $$

Rigas and Sebos^40^ show that the steel pipe should be considered fully flexible compared to the ground, as the flexibility ratio of ground to pipe stiffness is more than 100 times. The worst-case strains and stresses due to pipe explosions occur in bending and tensile strains parallel to the pipe axis and perpendicular to the circumferential stresses. These can be calculated by the following Eq. (28)^39^:

28 $$ \begin{aligned} q(x) & = c_{s} \rho_{L} V_{o} \\ \varepsilon_{b} & = \frac{{V_{o} \pi f_{v} D_{t} }}{{c_{s}^{2} }} \\ \varepsilon_{s} & = \frac{{V_{o} }}{{c_{l} }} \\ \varepsilon_{c} & = \frac{{V_{o} }}{{2c_{s} }} \\ \end{aligned} $$

where *V*_*0*_ is the peak particle velocity, *ε* is the strain, *b* is the bending direction, *S* is the stretching direction, *C* is the circumferential direction, *C*_*s*_ and *C*_*l*_ are the propagation velocities of shear and compressional waves, and *F*_*v*_ is the wave frequency.

By using the biaxial equation^41^, the circumferential and axial (longitudinal) stresses can be calculated from strains as follows.

29a $$ s_{l} = \frac{E}{{1 - \nu^{2} }}\left( {\varepsilon_{b} + \nu \varepsilon_{c} } \right) $$

29b $$ s_{c} = \frac{E}{{1 - \nu^{2} }}\left( {\varepsilon_{c} + \nu \varepsilon_{b} } \right) $$

**Figure 8:**
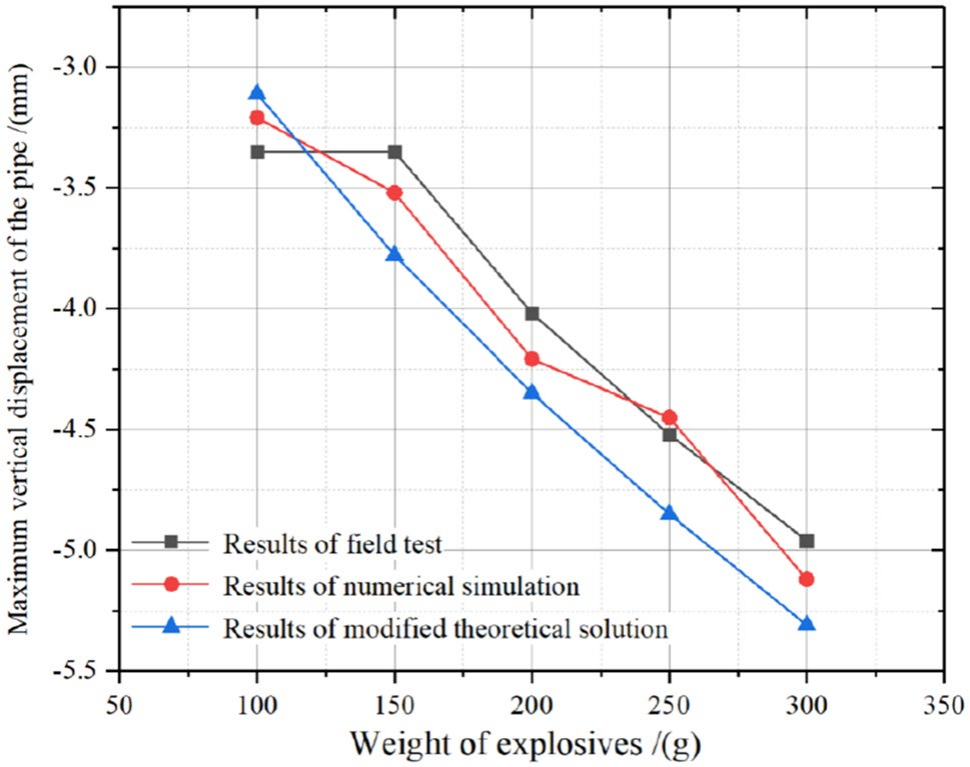
Comparison of results of the modified theoretical solution and field tests and numerical simulation.

Using Eq. (29b), we can obtain the stresses on the pipe from the surface blast load, which consists mainly of axial and circumferential stresses. Of these, it is the circumferential stresses that should apply to Timoshenko’s theory, so the Sc calculated in Eq. (29b) is substituted for q(x) and its value is brought into Eqs. (21)–(24), which allows the value of the longitudinal deformation of the existing pipe under surface blast loading to be solved in this way, as shown in Fig. 8. As can be seen in Fig. 8, the modified theoretical solution is close to the field test results and numerical simulation results, and the trend of both three curves is the same. At the same time, it can be seen from Fig. 8 that the maximum displacement of the pipe in the numerical simulation result is similar to that in the field test result, and with the increase of the weight of the explosive, the displacement of the pipe has little difference and fluctuates in a small range, while the theoretical solution is lower than the field test results, mainly because the theoretical equation calculation ignores the problems that can occur during wave propagation. Moreover, this problem is mainly related to the secondary dissipation of wave energy caused by reflection and refraction. But in any case, the overall error between the three is low and the maximum error does not exceed 12.9%. However, by comparing Fig. 7d, it can be seen that it is significantly improved, and its maximum error is less than 15%, so the modified theoretical solution can be used for the solution of the pipeline safety criterion model under surface explosive loads.

### Model for pipeline safety criteria under surface explosive loading

The safety of underground pipelines is influenced by many parameters, and damage criteria regarding pipelines can be divided into force yield damage and deformation damage^40,42–45^. In this paper, the damage criterion of deflection to span ratio is used to assess the extent of damage to the pipeline in surface explosive loading, as shown in Table 4.

**Table 4:** Damage criteria for different levels.

Damage level	Slight damage	Moderate damage	Severe damage	Collapse
Deflection-span ratio	< 1/200	1/200–1/100	1/100–1/50	> 1/50
Displacement/mm	< 5	5–10	10–20	> 20

**Figure 9:**
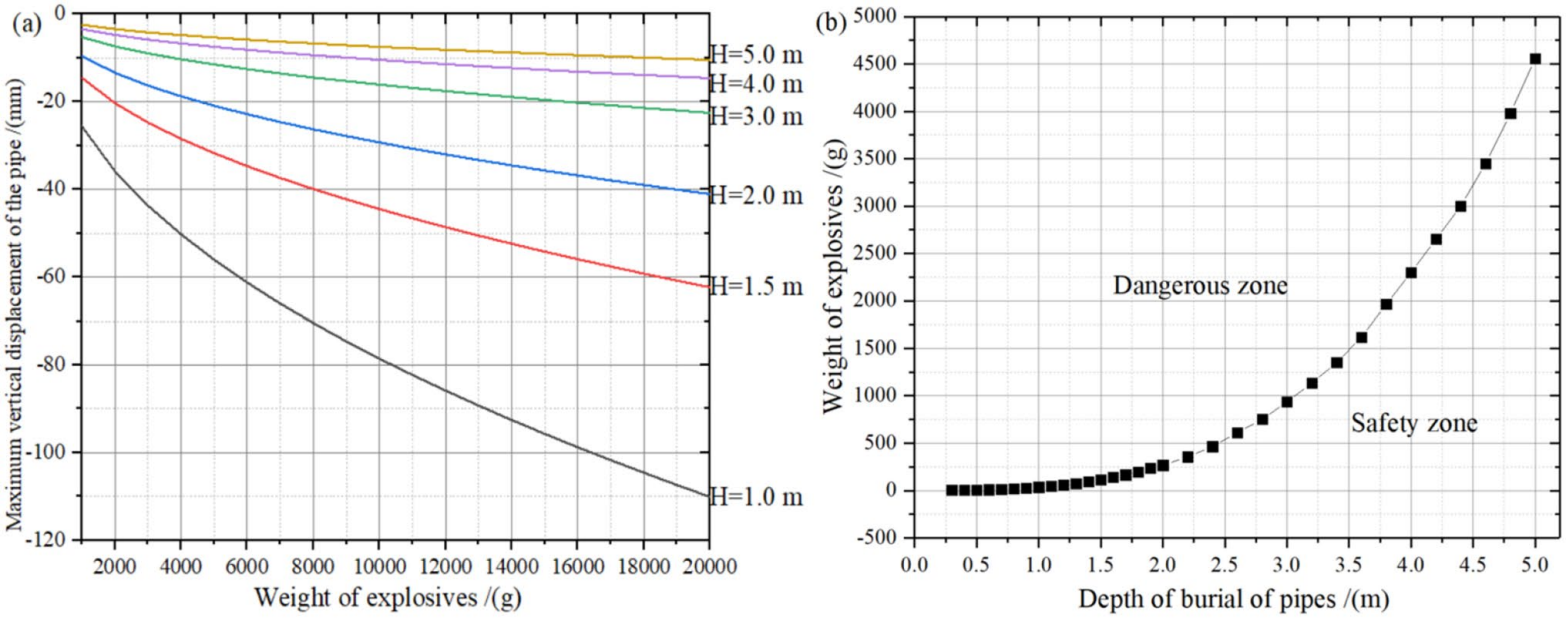
Change of maximum vertical displacement of the pipeline under surface explosive loads under different working conditions.

Through the validated theoretical calculation model, the pipeline safety criterion model under different surface explosive loads was derived, while the maximum vertical displacement change of the pipeline under surface explosive loads under different working conditions was studied, as shown in Fig. 9. Meanwhile, it is easy to notice that the main changes in Fig. 9 are the weight of the explosive above the pipe and the burial depth of the pipe. In addition, after fitting the curve where Fig. 9b is located, the prediction formula for the critical value of the weight of the explosive to prevent pipeline damage under different burial depths is obtained as shown in Eq. (30). In addition, it is not difficult to find through Fig. 9a that when the burial depth of the pipe is greater than or equal to 3 m, the maximum vertical displacement of the pipe varies slightly, and the displacement of the pipe decreases linearly with a small slope as the weight of the explosive increases. However, as the burial depth of the pipe decreases, the maximum vertical displacement of the pipe gradually increases. In addition, when the burial depth of the pipe is less than or equal to 2 m, the maximum vertical displacement of the pipe increases with the increase of the maximum weight of the explosive, and the function form of the fitting curve is the exponential function. This is due to the buffering effect of the soil on the blasting seismic waves generated by the surface explosives, and the fact that thicker soils contain more joints and fissures, which have an absorbing effect on the blasting seismic waves. Moreover, as the soil depth increases, the blasting seismic wave is constantly refracted and reflected and gradually decreases, thus resulting in a greater vertical maximum displacement of the pipe when the depth of burial is greater. What’s more, the critical weight of explosives to prevent damage to the pipeline increases exponentially with the increase in the depth of burial of the pipeline. Meanwhile, it is also interesting to note that when the depth of burial is less than or equal to 2 m, the critical weight of the explosive does not change much, but as the depth of burial increases, the critical weight of the explosive increases exponentially.

30 $$ Q_{c} = 39.353*H^{(2.8723)} + 100\quad \left( {R^{2} = 0.86} \right) $$

## Conclusion

The theoretical study of the deformation of pipelines by surface blast loads has been a hot topic of research at home and abroad. In this paper, combining Timoshenko beam theory and the elastic ratio theory of foundation stiffness to pipeline stiffness, the modified theoretical model in line with the actual situation in the field is obtained, and the deformation laws and safety guidelines of different types of pipelines under the action of surface blast loads are studied according to this theoretical model.

The model for calculating the pipe displacement without considering the flexible ratio theory of foundation stiffness to pipe stiffness is far from the numerical simulation results and field test results, while when applying the theoretical stresses in the Timoshenko beam, the original stress was replaced with circumferential stress, the modified theoretical solution is close to the field test results and numerical simulation results. Meanwhile, the validated theoretical calculation model resulted in a model of the pipeline safety criteria for different surface explosive loads, while the maximum vertical displacement of the pipeline increased exponentially with the weight of the explosive at different burial depths. More importantly, when the burial depth is greater than 2 m, the change in critical weight of the explosive varies dramatically with increasing burial depth, so it is recommended that for better protection against damage from surface blast loads, the recommended burial depth of the pipeline is at least 2 m.

The model proposed in this paper only considers the impact of the explosion load above the vertical surface of the pipeline on the pipeline displacement but does not consider the impact of explosives buried in the soil or different horizontal distances on the pipeline displacement prediction formula. Moreover, it provides a theoretical basis for the protection of pipelines’ underground blast loads and provides research ideas for the safe protection and design of pipelines.

## Data Availability

All data generated or analyzed during this study are included in this published article.

## References

[1] Li X, Zhang Y, Abbassi R (2021). Dynamic probability assessment of urban natural gas pipeline accidents considering integrated external activities. J Loss Prevent. Proc..

[2] Vasseghi A, Haghshenas E, Soroushian A (2021). Failure analysis of a natural gas pipeline subjected to landslide. Eng Fail. Anal..

[3] Zhu B, Jiang N, Zhou C (2021). Dynamic failure behavior of buried cast iron gas pipeline with local external corrosion subjected to blasting vibration. J Nat. Gas Sci. Eng..

[4] Qin G, Gong C, Wang Y (2021). A probabilistic-based model for predicting pipeline third-party hitting rate. Process. Saf. Environ..

[5] Tza B, Zheng LA, Liang ZA (2021). Safety assessment of buried natural gas pipelines with corrosion defects under the ground settlement. Eng. Fail. Anal..

[6] Rigas FP (2020). One-step estimation method and nomogram to predict safety distances of pressurized gas pipelines from blast sources. J Loss Prevent. Proc..

[7] Wu TY, Jiang N, Zhou CB (2021). Evaluate of anti-explosion for high-pressure gas steel pipeline subjected to ground explosion. J Constr. Steel. Res..

[8] Qiu J, Jiang B, Tang M (2021). Effect of different bend pipes on the propagation characteristics of premixed methane-air explosion in confined spaces. Geofluids.

[9] Global terrorism database. https://www.start.umd.edu/gtd/search/Results.aspx?search=&sa.x=54&sa.y=3. (2009–2022).

[10] Jiang N, Jia Y, Yao Y (2021). Experimental investigation on the influence of tunnel crossing blast vibration on upper gas pipeline. Eng. Fail. Anal..

[11] Zhao K, Jiang N, Zhou CB (2022). Dynamic behavior and failure of buried gas pipeline considering the pipe connection form subjected to blasting seismic waves. Thin Wall Struct..

[12] Qu Y, Li Z, Zhang R (2021). Dynamic performance prediction and influencing factors analysis of buried polyethylene pipelines under subsurface localized explosion. Int. J. Pres. Ves. Pip..

[13] Meng Q, Wu C, Li J (2021). A study of pressure characteristics of methane explosion in a 20 m buried tunnel and influence on structural behaviour of concrete elements. Eng. Fail. Anal..

[14] Zhang JL, Liang Z (2018). Buckling failure of a buried pipeline subjected to ground explosions. Process. Saf. Environ..

[15] Song KJ, Gao FY, Chen C (2016). Experimental and numerical studies on the deformation and tearing of x70 pipelines subjected to localized blast loading. Thin Wall Struct..

[16] Mishra B, Padhee PR, Singh RK (2020). Failure analysis and preventive measure proposition of self-flow castable blow pipe of blast furnace tuyere stock for enhancing reliability. Trans Indian Inst. Met..

[17] Zhang G (2020). Experimental study on shock wave propagation of the explosion in a pipe with holes by high-speed schlieren method. Shock Vib..

[18] Bambach MR (2008). Behaviour and design of aluminium hollow sections subjected to transverse blast loads. Steel Constr..

[19] Abedi AS, Hataf N, Ghahramani A (2016). Analytical solution of the dynamic response of buried pipelines under blast wave. Int. J. Rock Mech. Min..

[20] Olarewaju AJ, Kameswara NSV, Mannan RMA (2010). Response of underground pipes due to blast loads by simulation—An overview. Electron. J. Geotech. Eng..

[21] Mirzaei M, Najafi M, Niasari H (2015). Experimental and numerical analysis of dynamic rupture of steel pipes under internal high-speed moving pressures. Int. J. Impact Eng..

[22] Zhang Z, Zhou C, Remennikov A (2021). Dynamic response and safety control of civil air defense tunnel under excavation blasting of subway tunnel. Tunn. Undergr. Space Technol..

[23] Yin YP, Wang LQ, Zhang WG, Dai ZW (2022). Research on the collapse process of a thick-layer dangerous rock on the reservoir bank. Bull. Eng. Geol. Environ..

[24] Ma JW, Wang YK, Niu XX, Jiang S, Liu ZY (2022). A comparative study of mutual information-based input variable selection strategies for the displacement prediction of seepage-driven landslides using optimized support vector regression. Stoch. Environ. Res. Risk A.

[25] Sari A, Sayin B, Khiavi MP (2021). A methodology to prevent process piping failures during vapor cloud explosions. Int. J. Pres. Ves. Pip..

[26] Parviz M, Aminnejad B, Fiouz A (2017). Numerical simulation of dynamic response of water in buried pipeline under explosion. KSCE J. Civ. Eng..

[27] Qi S, Zhi X, Fan F (2020). Propagation behaviour of a hemispherical blast wave on a dome roof. Eng. Struct..

[28] Trivino, L., Mohanty, B. & Munjiza, A. Seismic radiation patterns from cylindrical explosive charges by combined analytical and combined finite-discrete element methods. In: Proceedings of 9th International Symposium on Rock Fragmentation by Blasting (FRAGBLAST’9) (ed Sanchidrian) 415–426 (Taylor & Francis, 2009).

[29] Blair DP (2003). A fast and efficient solution for wave radiation from a pressurized blasthole. Fragblast Int. J. Blast. Fragm..

[30] Duvall WI (1953). Strain-wave shapes in rock near explosions. Geophysics.

[31] Esen S, Onederrax I, Bilgin HA (2003). Modelling the size of the crushed zone around a blasthole. Int. J. Rock Mech. Min..

[32] Zhang Z, Huang M, Wang W (2013). Evaluation of deformation response for adjacent tunnels due to soil unloading in excavation engineering. Tunn. Undergr. Space Technol..

[33] Hrytsyna, O. Timoshenko elastic and electroelastic beam models incorporating the local mass displacement effect. In: International Conference on Computational & Experimental Engineering and Sciences. 303–322 (Springer, Cham, 2021).

[34] Kiendl J, Auricchio F, Reali A (2018). A displacement-free formulation for the Timoshenko beam problem and a corresponding isogeometric collocation approach. Meccanica.

[35] Steel, B., July, P. Guidelines for the Design of Buried Steel Pipe. (2005).

[36] Zhang J, Liang Z, Han CJ (2015). Numerical simulation of mechanical behavior of buried pipeline impacted by perilous rock. Mechanika.

[37] GB T 9711–2011. Steel pipes for pipeline transmission systems in the oil and gas industry (2001).

[38] Mokhtari M, Nia AA (2015). A parametric study on the mechanical performance of buried X65 steel pipelines under subsurface detonation. Arch. Civ. Mech. Eng..

[39] Dowding HC (1996). Construction Vibrations.

[40] Rigas F, Sebos I (1998). Shortcut estimation of safety distances of pipelines from explosives. J. Transp. Eng..

[41] Timoshenko SP, Woinowski-Krieger S (1959). Theory of Plates and Shells.

[42] McVay, M. Spall damage of concrete structures. US Army Corps of Engineers Waterways Experiment Station. (1988).

[43] Fallah AS, Louca LA (2007). Pressure-impulse diagrams for elastic-plastic-hardening and softening single-degree-of-freedom models subjected to blast loading. Int. J. Impact Eng..

[44] Mussa MH, Mutalib AA, Hamid R, Naidu SR, Radzi NAM, Abedini M (2017). Assessment of damage to an underground box tunnel by a surface explosion. Tunn. Undergr. Space Technol..

[45] Shi YC, Li ZX, Hao H (2010). A new method for progressive collapse analysis of RC frames under blast loading. Eng. Struct..

